# Improved risk prediction of chemotherapy‐induced neutropenia—model development and validation with real‐world data

**DOI:** 10.1002/cam4.4465

**Published:** 2021-12-03

**Authors:** Mikko S. Venäläinen, Eetu Heervä, Outi Hirvonen, Sohrab Saraei, Tomi Suomi, Toni Mikkola, Maarit Bärlund, Sirkku Jyrkkiö, Tarja Laitinen, Laura L. Elo

**Affiliations:** ^1^ Turku Bioscience Centre University of Turku and Åbo Akademi University Turku Finland; ^2^ Department of Oncology Turku University Hospital and FICAN West Turku Finland; ^3^ University of Turku Turku Finland; ^4^ Department of Clinical Oncology University of Turku Turku Finland; ^5^ Palliative Center Turku University Hospital Turku Finland; ^6^ Tays Research Services Clinical Informatics Team Tampere University Hospital and University of Tampere Tampere Finland; ^7^ Department of Oncology Tays Cancer Centre Tampere University Hospital Tampere Finland; ^8^ Faculty of Medicine and Health Technology Tampere University Tampere Finland; ^9^ Department of Pulmonary Medicine University of Turku and Turku University Hospital Turku Finland; ^10^ Administration Center Tampere University Hospital and University of Tampere Tampere Finland; ^11^ Institute of Biomedicine University of Turku Turku Finland

**Keywords:** chemotherapy, clinical decision support, granulocyte colony‐stimulating factor, machine learning, neutropenia

## Abstract

**Background:**

The existing risk prediction models for chemotherapy‐induced febrile neutropenia (FN) do not necessarily apply to real‐life patients in different healthcare systems and the external validation of these models are often lacking. Our study evaluates whether a machine learning‐based risk prediction model could outperform the previously introduced models, especially when validated against real‐world patient data from another institution not used for model training.

**Methods:**

Using Turku University Hospital electronic medical records, we identified all patients who received chemotherapy for non‐hematological cancer between the years 2010 and 2017 (*N* = 5879). An experimental surrogate endpoint was first‐cycle neutropenic infection (NI), defined as grade IV neutropenia with serum C‐reactive protein >10 mg/l. For predicting the risk of NI, a penalized regression model (Lasso) was developed. The model was externally validated in an independent dataset (*N* = 4594) from Tampere University Hospital.

**Results:**

Lasso model accurately predicted NI risk with good accuracy (AUROC 0.84). In the validation cohort, the Lasso model outperformed two previously introduced, widely approved models, with AUROC 0.75. The variables selected by Lasso included granulocyte colony‐stimulating factor (G‐CSF) use, cancer type, pre‐treatment neutrophil and thrombocyte count, intravenous treatment regimen, and the planned dose intensity. The same model predicted also FN, with AUROC 0.77, supporting the validity of NI as an endpoint.

**Conclusions:**

Our study demonstrates that real‐world NI risk prediction can be improved with machine learning and that every difference in patient or treatment characteristics can have a significant impact on model performance. Here we outline a novel, externally validated approach which may hold potential to facilitate more targeted use of G‐CSFs in the future.

## Graphical abstract

There are several risk prediction models for chemotherapy‐induced neutropenia, but the existing models may not always apply to real‐life patients in different healthcare systems. A novel machine learning‐based model improved neutropenic infection risk prediction as compared to previously introduced models. Our validated model may facilitate more targeted use of granulocyte colony‐stimulating factors in the future.

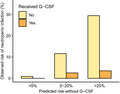

**Novelty and Impact**

There are several risk prediction models for chemotherapy‐induced neutropenia, but the existing models may not always apply to real‐life patients in different healthcare systems. A novel machine learning‐based model was developed to predict neutropenic infection risk in cancer patients. The model performance was externally validated in an independent cohort, outperforming two previously introduced conventional models. In the future, our model may facilitate more targeted use of granulocyte colony‐stimulating factors.

## 1 INTRODUCTION

Prophylactic granulocyte colony‐stimulating factors (G‐CSFs) can be used to shorten neutropenia duration and prevent febrile neutropenia (FN), which are major dose‐limiting and resource‐intensive complications during cancer chemotherapy. Meta‐analyses show that use of G‐CSFs significantly reduces the risk of FN, with relative risks of 0.27–0.51 compared to patients with no G‐CSF prophylaxis.^1^, ^2^ However, risk assessment based on real‐world settings are sparse.^3^

Significant underuse of G‐CSFs in high‐ and intermediate‐risk regimens, and overuse in low‐risk regimens, have been reported both in the EU^4^, ^5^ and in the US.^6^, ^7^, ^8^ G‐CSFs contribute also significantly to increased healthcare costs.^9^ Therefore, selective use of G‐CSFs based on predetermined FN risk is recommended to optimize the use of healthcare resources.^10^, ^11^

European and North American guidelines recommend the use of prophylactic G‐CSF based on the predetermined risk of FN, classified as low, intermediate, or high, depending on the chemotherapy regimen.^10^, ^11^ Prophylactic G‐CSFs are recommended for high risk but not for low‐risk regimens. For intermediate‐risk regimens, a history of prior neutropenia or leukopenia, age >65 years, presence of comorbid conditions, advanced stage of disease (especially to bone marrow), poor performance status, female sex, and low hemoglobin support the use of prophylactic G‐CSFs.^10^, ^11^, ^12^, ^13^, ^14^

Risk prediction models for neutropenic complications based on retrospective data from the US^14^, ^15^ or on prospective cohorts and trial settings in Europe^12^, ^16^ or tailored for specific cancer type^17^ have been introduced. Model by Lyman et al. is commonly used, which is based on 3760 US patients with different types of cancer treated during 2002–2006^14^ and has subsequently been re‐evaluated in a larger population.^15^ Despite the available models, they have not been typically externally validated against data from institutions other than the one used for model training.^17^ In the development of any risk prediction model, external validation is of great importance to verify the generalizability of the model to patients outside the training cohort and should always be done before its implementation and wider use in a clinical setting.^18^

In addition to the lack of external validation, the development of previous models has typically relied on conventional modeling strategies whose performances might be improved with machine learning‐based modeling approaches.^19^ For example, the widely used model by Lyman et al. is a multivariable logistic regression model developed by applying a stepwise variable selection procedure^14^ in which the variable selection process is known to be more unstable compared to penalized approaches and can magnify problems associated with model overfitting.^19^, ^20^ The recently re‐evaluated version of this model introduced by Li et al. also uses the same modeling approach and same variables as the original study but with few variable‐related modifications made based on clinical and numerical rationale.^15^

The aim of the present study was to evaluate if a machine learning‐based risk prediction model could outperform previously introduced models by Lyman et al. and Li et al.^14^, ^15^ for neutropenic complications in real‐world patient data. To avoid overfitting and to verify model generalizability, we carried out external validation against independent data from another university hospital.

## 2 MATERIAL AND METHODS

### 2.1 Patients

We gathered comprehensive clinical data on all patients who received intravenous chemotherapy between January 2010 and December 2017 at Turku University Hospital, Finland, covering the population of 480 000 inhabitants in Southwest Finland. This data included the patient's sex, age, time of death, weight, height, body temperatures, given diagnoses (ICD‐10 codes), electronic prescriptions, intravenous chemotherapies and monoclonal antibodies, G‐CSF therapies, laboratory values, and hospital in‐ and outpatient visits.

**FIGURE 1:**
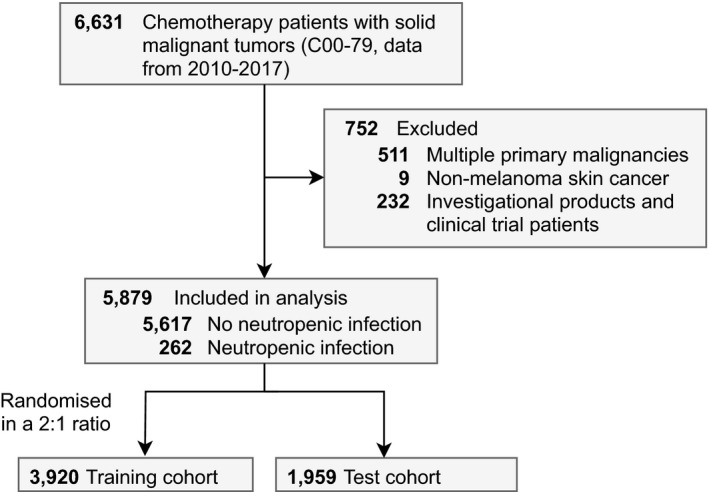
Selection of patients into the Turku University Hospital cohort

All patients aged at least 18 years with ICD‐10 code C00‐79 before receiving their first dose of intravenous chemotherapy, either in curative or palliative regimens, were included (Figure 1). Patients treated with investigational products or non‐chemotherapy regimens such as immune checkpoint inhibitors (232 patients), patients with nonmelanoma skin cancer (9 patients), and patients having multiple primary malignancies (511 patients) were excluded. This left a total of 5879 patients in the analyses. For model training and internal testing, the data were randomized into separate training (*N* = 3920, two thirds of the data) and test (*N* = 1959, one third of the data) cohorts.

Using the same protocol, we collected an independent validation cohort from Tampere University Hospital with the catchment population of 515 100 inhabitants. In total, 4594 patients treated between January 2014 and June 2019 were identified.

The data gathering and analysis were performed with research permissions granted by institutional review boards of Turku and Tampere University Hospitals. Additional information on data gathering and confidentiality can be found in the Supplementary Material.

### 2.2 Study endpoint and candidate predictors

Febrile neutropenia is a classical endpoint in neutropenia studies, but the definition of FN varies depending on the source.^14^, ^21^ We designed neutropenic infection (NI) as primary endpoint, which was defined as grade IV neutropenia (absolute neutrophil count (ANC) <0.5 × 10^9^/l according to Common Terminology Criteria for Adverse Events (CTCAE) version 4.0) within 14 days of the first chemotherapy infusion combined with subsequent serum C‐reactive protein (CRP) level >10 mg/l within 5 days (of neutropenia). Patients who did not undergo any laboratory measurements within 14 days of the first chemotherapy infusion or failed to fulfill either of the two criteria were considered patients without NI. To further verify the selected primary study endpoint, the occurrence of NI was compared with admissions to any tertiary care hospital ward during the NI episode. We also tested FN as an endpoint, defined as body temperature ≥38°C and ANC <1.0 × 10^9^/l according to CTCAE version 4.0 but ignoring the required 1‐hour limit for fever. We focused on the first‐cycle of chemotherapy when the patients are at highest risk of developing neutropenia.^12^, ^14^

The candidate predictors for estimating the risk of NI are provided in Table 1 and in the Supplementary Material with additional details.

**TABLE 1:** Characteristics of the Turku University Hospital cohort according to the occurrence of neutropenic infection during the first round of chemotherapy

	Neutropenic infection	Neutropenic infection	*p* value^b^
	No	Yes	
	*N* = 5617	*N* = 262	
Demographics			
Sex, *N* (%)			<0.001
Male	2228 (40)	41 (16)	
Female	3389 (60)	221 (84)	
Age, *N* (%)			<0.001
<40	260 (5)	20 (8)	
40–65	2691 (48)	173 (66)	
>65	2658 (47)	69 (26)	
Mean BMI, kg/m² (standard deviation)	26.6 (5.4)	26.3 (4.9)	0.7
Mean body surface area, m² (standard deviation)	1.9 (0.2)	1.8 (0.2)	0.009
Use of prophylactic G‐CSFs, *N* (%)	306 (5)	8 (3)	0.1
Comorbidities [ICD−10], *N* (%)			
COPD [J44, J96]	282 (5)	13 (5)	1.0
Coronary heart disease [I25]	369 (7)	10 (4)	0.1
Diabetes [E10‐E14]	524 (9)	10 (4)	0.003
Heart failure [I50]	123 (2)	6 (2)	1.0
Renal impairment [N17‐N19]	73 (1)	3 (1)	1.0
Liver failure [K70‐K75]	64 (1)	3 (1)	1.0
Rheumatoid arthritis [M05‐M07]	118 (2)	4 (2)	0.7
Ulcer disease [K25‐K27]	89 (2)	2 (1)	0.4
Metastatic disease			
C77‐C79 detected, *N* (%)	791 (14)	17 (6)	<0.001
Laboratory test results, mean (standard deviation)			
Absolute neutrophil count [×10^9^/l]	4.4 (2.4)	3.6 (2.8)	<0.001
Alanine aminotransferase [U/l]	29.2 (31.1)	27.5 (21.4)	0.7
Alkaline phosphatase [U/l]	100.0 (126.8)	80.1 (64.0)	<0.001
Average red blood cell size [fl]	89.8 (5.3)	90.4 (4.7)	0.2
Blood hematocrit [%]	39.7 (4.1)	40.2 (3.4)	0.05
Blood hemoglobin [g/l]	131.4 (15.3)	133.8 (12.8)	0.03
Hemoglobin amount per red blood cell [pg]	29.8 (2.3)	30.2 (1.9)	0.01
Leukocyte count [×10^9^/l]	7.4 (5.4)	6.6 (3.1)	<0.001
Plasma bilirubin [μmol/l]	9.5 (8.5)	9.4 (5.6)	0.2
Plasma potassium [mmol/l]	4.0 (0.4)	4.1 (0.3)	0.9
Plasma sodium [mmol/l]	140.3 (3.5)	140.8 (3.7)	0.002
Red blood cell count [×10^12^/l]	4.4 (0.5)	4.5 (0.4)	0.3
Serum creatinine [μmol/l]	73.1 (19.8)	71.7 (16.8)	0.5
Thrombocyte count [×10^9^/l]	300.0 (104.8)	267.9 (69.5)	<0.001
Planned relative dose intensity, *N* (%)			<0.001
< 85%	1103 (25)	24 (10)	
≥ 85%	3224 (75)	205 (90)	
Intravenous treatment regimens, *N* (%)			
Alkylating agents	880 (16)	15 (6)	<0.001
Anthracyclines	909 (16)	14 (5)	<0.001
Antimetabolites	2591 (46)	40 (15)	<0.001
Antitumor antibiotics	126 (2)	3 (1)	0.3
Monoclonal antibodies	439 (8)	81 (31)	<0.001
Platinum	2154 (38)	40 (15)	<0.001
Taxanes	1733 (31)	199 (76)	<0.001
Topoisomerase inhibitors	419 (7)	28 (11)	0.07
Vinca alkaloids	188 (3)	3 (1)	0.07
Cancer group [ICD−10], *N* (%)			<0.001
Breast [C50]	1845 (33)	204 (78)	
Central nervous system [C70‐72]	80 (1)	0 (0)	
Colorectal [C18‐20]	692 (12)	6 (2)	
Female reproductive [C51‐57]	436 (8)	3 (1)	
Gastric [C15‐16]	241 (4)	0 (0)	
Head and neck [C00‐14, C30‐32]	376 (7)	0 (0)	
Lung, non‐small cell [C33‐35]	530 (10)	20 (8)	
Lung, small cell [C33‐35]	161 (3)	11 (4)	
Melanoma [C43]	49 (1)	0 (0)	
Other gastrointestinal [C17, C21, C22, C26]	78 (1)	1 (0)	
Pancreas and gallbladder [C23‐25]	301 (5)	2 (1)	
Prostate [C61]	286 (5)	2 (1)	
Sarcoma [C40‐41, C46‐49]	69 (1)	5 (2)	
Testicular [C62]	93 (2)	3 (1)	
Urinary tract [C65‐68]	261 (5)	3 (1)	
Other^a^	119 (2)	2 (1)	

^a^ Category includes all remaining ICD‐10 codes from C00‐79.

^b^ Comparisons between the groups either having or not having NI were tested using the Mann–Whitney test for continuous variables and the chi‐squared test or Fisher's exact test (*N* < 5) for categorical variables.

### 2.3 Statistical analysis and model development

We applied penalized logistic regression with least absolute shrinkage and selection operator (Lasso) penalty to the training cohort to construct a multivariable model for predicting the individualized risk of NI. To identify the most influential predictors and to account for model variability due to random subsampling, the model construction was performed iteratively in multiple steps similarly as before.^22^, ^23^, ^24^ In previous studies, this approach has led to models with fewer variables but retaining the same prediction accuracy as the more complex models. During model development and validation, only patients with complete data for the selected predictors were used.

The performance of the Lasso model was compared against previously introduced model for multiple cancer types by Lyman et al.^14^ as well as the revised version of the Lyman model introduced by Li et al.^15^ (Table S1). The comparisons were done using only those cancer types that were shared by all three studies (breast, ovarian, colorectal, small cell, and non‐small cell lung cancer) and patients for whom all risk estimates could be determined. Furthermore, patients who received G‐CSFs were excluded from this comparison since they were not included in the development of Li model. Finally, we compared the performance of the Lasso model also with a model developed using the conventional stepwise variable selection procedure.

All statistical analyses and modeling were carried out using the R statistical computing environment version 3.4.3 (R Core Team, 2016. R: A language and environment for statistical computing. R Foundation for Statistical Computing, Vienna, Austria. URL https://www.R‐project.org/). For penalized regression, implementation available in the R package *glmnet* (version 2.0–16)^25^ was used. The discrimination performances of the risk assessment models were evaluated in terms of area under the receiver operating characteristic curve (AUROC) and compared using the DeLong test^26^ implemented in the R package *pROC* (version 1.12.1). Comparisons between the groups either having or not having NI were tested using the Mann–Whitney test for continuous variables and the chi‐squared test or Fisher's exact test (*N* < 5) for categorical variables. The level of significance was set at *p* < 0.05. Additional details on model development and statistical analyses can be found in the Supplementary Material.

## 3 RESULTS

### 3.1 Study populations

In total, the Turku University Hospital cohort consisted of 5879 patients (Table 1). Among these, NI occurred in 262 (4%) of the patients, of whom 225 (86%) were also subsequently admitted to a hospital ward. Out of 5879 patients, 314 (5%) received G‐CSFs as primary prophylaxis, of whom eight (3%) developed NI. The patients who had NI were typically women (*p* < 0.001) with breast cancer (*p* < 0.001) treated with higher relative dose intensity (RDI) (*p* < 0.001) and had lower levels of blood ANC (*p* < 0.001), leukocytes (*p* < 0.001), and thrombocytes (*p* < 0.001) in the beginning of the treatment (Table 1). Despite the lower ANC and thrombocyte counts, for nearly all patients (98%) these were above the lower limit of the reference (>1.5 × 10^9^/l for ANC and >150 × 10^9^/l for thrombocyte count).

The validation cohort from Tampere University Hospital included 4594 patients who showed similar distributions in age, sex, comorbidities, cancer types, and RDI compared to the Turku University Hospital cohort (Table S2). The increased risk of NI associated with lower ANC and thrombocyte counts, however, were not observed in the validation cohort.

### 3.2 Model development and validation

Of the more than 30 variables included (Table 1), a subset of 10 variables (Table 2) were selected by the Lasso model for accurate NI risk predictions in the training cohort (AUROC 0.87, 95% confidence interval [CI] 0.84–0.89). The selected model showed similar performance also in the internal test cohort indicating good generalizability (AUROC 0.85, 95% CI 0.80–0.90). As expected, use of prophylactic G‐CSFs and reduced RDI were among the most influential predictors decreasing NI risk (Table 2). Intravenous treatment regimens involving antimetabolites demonstrated also decreased NI risk compared to other treatment regimens. Of all cancer types, breast cancer and sarcoma patients showed increased NI risk. The other variables increasing NI risk were use of taxanes alone or in combination with monoclonal antibodies, use of topoisomerase inhibitors, and low pre‐treatment ANC and thrombocyte counts. The majority of taxanes used were docetaxel 80 mg/m^2^ Q3W to treat breast cancer (65%), docetaxel 50 mg/m^2^ Q2W to treat metastatic castration‐resistant prostate cancer (14%), and paclitaxel to treat gynecological cancers (11%). We observed that among patients treated with taxanes at full RDI, NI risk was 12%, compared to 3% in those treated with reduced RDI.

**TABLE 2:** Coefficients and covariates in the Lasso risk assessment model for the occurrence of neutropenic infection during the first round of chemotherapy in the training cohort

Covariate	Coefficient^a^	Effect	OR (95% CI)^b^	*p* value
Intercept	0.477	Baseline risk	–	–
Cancer type				
Breast cancer	2.361	Increased risk	7.20 (5.39–9.77)	<0.001
Sarcoma	3.694	Increased risk	1.57 (0.55–3.55)	0.34
Laboratory test results				
Neutrophil count [×10^9^/l] (per ln increase)	−0.282	Decreased risk	0.41 (0.31–0.56)	<0.001
Thrombocyte count [×10^9^/l] (per ln increase)	−0.966	Decreased risk	0.44 (0.30–0.64)	<0.001
Treatment regimen				
Use of taxanes	1.262	Increased risk	7.10 (5.35–9.54)	<0.001
Combined use of taxanes and monoclonal antibodies	0.871	Increased risk	8.53 (6.37–11.35)	<0.001
Use of topoisomerase inhibitors	3.305	Increased risk	1.49 (0.97–2.19)	0.05
Use of antimetabolites	−0.787	Decreased risk	0.21 (0.15–0.29)	<0.001
Actions to reduce risk				
Use of G‐CSF	−1.780	Decreased risk	0.55 (0.25–1.04)	0.10
Relative dose intensity <85%	−0.814	Decreased risk	0.34 (0.22–0.51)	<0.001

^a^ Coefficients indicate the impact of a 1‐unit change in a predictor variable on the response variable when the other predictors are held constant.

^b^ The odds ratios (OR) and corresponding *p* values were estimated separately using univariable logistic regression without penalization. CI denotes confidence interval.

Overall, the Lasso model predicted NI risk with high accuracy in the Turku University Hospital cohort (AUROC 0.86, 95% CI 0.84–0.89) as well as with fair accuracy in the Tampere University Hospital validation cohort (AUROC 0.73, 95% CI 0.69–0.77). For cancer types included in the development of previous models, the Lasso model significantly outperformed the previous models in both Turku University Hospital (Lyman model AUROC 0.47, *p* < 0.001; Li model AUROC 0.78, *p* < 0.001) and Tampere University Hospital cohorts (Lyman model AUROC 0.53, *p* < 0.001; Li model AUROC 0.70, *p* = 0.01), with AUROCs of 0.84 and 0.75, respectively (Table 3). The discrimination performance of the model developed using the conventional stepwise variable selection procedure was comparable to the Lasso model in the Turku University Hospital cohort but significantly worse in the Tampere University Hospital cohort (Supplementary Material).

**TABLE 3:** Comparison of the discrimination performances of the developed Lasso model, Lyman model,^14^ and Li model^15^ in Turku University Hospital and Tampere University Hospital validation cohorts for the occurrence of neutropenic infection and febrile neutropenia

Model	Turku University Hospital (*N *= 2101)				Tampere University Hospital Validation cohort (*N *= 1937)	
	Outcome: Neutropenic infection		Outcome: Febrile neutropenia		Outcome: Neutropenic infection	
	AUROC (95% CI)	*p* value^a^	AUROC (95% CI)	*p* value^a^	AUROC (95% CI)	*p* value^a^
Lasso	0.84 (0.81–0.86)	–	0.77 (0.73–0.81)	–	0.75 (0.69–0.77)	–
Lyman	0.47 (0.43–0.50)	< 0.001	0.50 (0.46–0.54)	< 0.001	0.53 (0.47–0.59)	< 0.001
Li	0.78 (0.75–0.80)	< 0.001	0.73 (0.70–0.76)	0.007	0.70 (0.66–0.74)	0.01

Note

Abbreviations: AUROC, area under the receiver operating characteristic curve; CI, confidence interval.

^a^ *p* values are reported for comparisons with the Lasso model.

In Turku University Hospital cohort, 56% of the patients with NI also met the criteria for FN. Overall, FN was observed in 221 (4%) of patients. When applied to predict the potential FN cases, the Lasso model significantly outperformed (AUROC 0.77) the previously introduced Lyman (AUROC 0.50, *p* < 0.001) and Li models (AUROC 0.73, *p* = 0.007) (Table 3).

### 3.3 Assessing the effect of G‐CSFs on predicted NI risk

**FIGURE 2:**
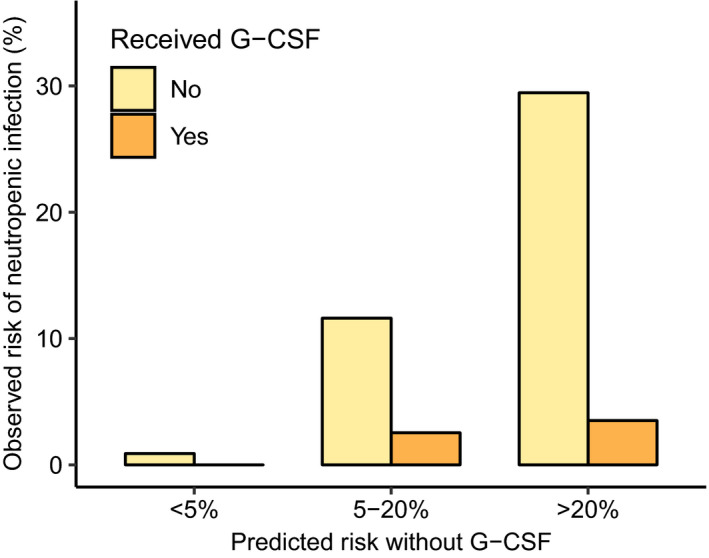
Observed rates of NI (y‐axis) for patients with and without G‐CSFs in different categories of predicted risk (x‐axis) in the Turku University Hospital cohort. The risk of NI was determined only for patients with complete baseline information for variables included in the Lasso risk assessment model (*N* = 3861)

We observed that, especially in the Turku University Hospital cohort, the higher the predicted risk of NI, the greater benefits from G‐CSFs can be expected in terms of reducing the risk (Figure 2). Among patients with the highest predicted NI risk (>20%), the observed NI rate was 29% (79 out of 270 patients) without the use of G‐CSF but roughly 3% when G‐CSF was used (2 out of 59 patients). The proportion of patients receiving G‐CSFs was also highest in this category (18%) compared to categories with predicted risk of 5–20% or <5% with 116 out of 850 patients (14%) or 52 out of 2502 patients (2%) receiving G‐CSFs, respectively. A practical example on how to apply the fitted Lasso model to evaluate the effect of G‐CSFs on NI risk is provided in the Supplementary Material.

## 4 DISCUSSION

The decision whether to use prophylactic G‐CSFs during chemotherapy is a daily question in oncological practice. Our study introduces a novel machine learning‐based model to predict NI risk when initiating chemotherapy for cancer patients. The Lasso model uses a limited number of routinely used variables and showed excellent capabilities in predicting NI risk in both training and external validation real‐world data cohorts, indicating good model generalizability.

The Lasso model outperformed both previously introduced models as well as a model based on stepwise variable selection procedure in predicting NI risk with even fewer required input variables suggesting that machine learning‐based modeling can improve both usability and predictive performance over more conventional approaches. This finding held true also in the validation cohort. Lack of external validation is a common problem in neutropenia prediction studies, limiting their implementation in clinical practice.

The improved performance is explained by the different input variables resulting from the applied iterative variable selection procedure used successfully also before.^22^, ^23^, ^24^ Alternatively, poorly adjusted regression coefficients due to inherent differences between patient populations and local healthcare practices or overfitting may explain the worse performance of previous models. For example, the Lyman model had drastically different coefficients for different treatment regimens compared to Lasso and Li models (Table 2, Table S1) which might explain its underperformance. This highlights also the importance of model validation in the target population before use.

The most common clinically monitored neutropenic complication is FN, for which several different diagnostic criteria can be found in the literature.^10^, ^14^, ^21^ However, FN was a challenging outcome measure in a real world, retrospective study setting, as comprehensive body temperature information is usually unavailable. Body temperature is not always documented or unreliable due to fever‐lowering medication. Therefore, we chose CRP (>10 mg/l) accompanied with severe neutropenia (ANC <0.5 × 10^9^/l) as the primary endpoint. In clinical practice, it is considered that these laboratory values are always available when initiating antimicrobial treatment, even if the body temperature is below 38.0°C. Notably, the majority (86%) of the patients identified as having NI were also admitted to a hospital ward, supporting the validity of our primary endpoint. The occurrence of first‐cycle NI (4%) was close to the occurrence of first‐cycle FN reported in the literature.^14^, ^15^ We also tested our risk assessment model for predicting FN, and observed a high level of accuracy, thus supporting the utility of our model for both FN and NI risk prediction.

Of the more than 30 risk factors studied, the Lasso model eventually required only a subset of 10 variables. As expected, the use of G‐CSFs showed a similar risk‐reducing effect against neutropenic complications as reported previously.^1^, ^2^, ^14^ Overall, patients receiving taxanes, with or without monoclonal antibodies to treat breast cancer formed the largest group of patients with increased NI risk. This is line with a recent meta‐analysis that demonstrated a significantly increased risk of FN with the use of trastuzumab,^27^ the most commonly used monoclonal antibody in our study cohort. The second risk group identified consisted of heterogeneous sarcoma patients, often treated with aggressive chemotherapy. Topoisomerase inhibitors, such as etoposide used in small‐cell lung cancer and irinotecan used in gastrointestinal cancers, increased risk of NI. However, antimetabolites including fluorouracil commonly used in several types of cancer, were associated with reduced relative NI risk. These chemotherapy regimens remained as important predictors for NI regardless of the RDI used.

Reduced RDI, another mechanism to reduce neutropenia risk along with G‐CSFs, was also identified as an important variable and could explain why age or comorbidities did not influence the Lasso model. This further reflects the oncology practice in Finland, where older and comorbid patients seem to be treated with reduced doses of chemotherapy. The risk‐reducing effect of reduced RDI was consistent with previous reports.^14^, ^15^ Finally, among all the pre‐treatment laboratory test results, only lower ANC and thrombocyte counts were associated with elevated NI risk in the final model. Similar effects of ANC and thrombocyte counts on the risk of neutropenic complications have been reported also before.^14^, ^28^ Lower thrombocyte count may reflect bone marrow dysfunction and could therefore be linked to simultaneous leukopenia, but this was not observed in the validation cohort and should be interpreted with caution.

In conclusion, morbidity due to neutropenic complications affects the patient's quality of life, creates substantial costs, and may even threaten the outcome of cancer treatment if the treatment schedule is postponed because of infection. To improve the targeted use of prophylactic G‐CSFs, well‐calibrated risk models applicable to real‐world data are needed. Here, we demonstrate that risk prediction of neutropenic complications can be improved with machine learning and that even previously validated models do not necessarily lead to correct predictions in all patient populations. Our novel machine learning‐based model outperformed both previously introduced models and can be easily applied to identify individuals at high risk of neutropenic complications especially in countries with similar clinical practices. These findings were confirmed in an external validation cohort thus supporting the generalizability and clinical applicability of our model. Overall, the presented method holds potential for avoiding resource‐intensive and life‐threatening neutropenic complications and could facilitate the proper use of G‐CSFs in the future.

## CONFLICT OF INTEREST

The authors declare no conflict of interest.

## AUTHOR CONTRIBUTIONS

Sirkku Jyrkkiö, Tarja Laitinen and Laura L. Elo contributed to conceptualization and reviewing, and editing the manuscript. Mikko S. Venäläinen and Toni Mikkola were responsible for formal analysis and visualization. Mikko S. Venäläinen, Sohrab Saraei, and Tomi Suomi contributed to methodology. Toni Mikkola performed external validations. Mikko S. Venäläinen and Eetu Heervä wrote the original draft of the manuscript. Eetu Heervä, Outi Hirvonen, Maarit Bärlund, Sirkku Jyrkkiö, and Tarja Laitinen interpreted the data and reviewed the manuscript critically for important intellectual content. Tarja Laitinen and Laura L. Elo provided resources for the project. Laura L. Elo was responsible for funding acquisition and supervised the project. All authors read and approved the final manuscript.

## ETHICAL STATEMENT

The requirements for ethical review in Finland are stipulated primarily in the Medical Research Act (488/1999, as amended) and the Act of the Medical Use of Human Organs, Tissues, and Cells (101/2001 as amended). Ethical review is statutorily required for interventional medical research and in some circumstances for studies on human organs, tissues, or cells. According to Finnish legislation, no ethical assessment or approval by an independent review board is mandatory for register studies, meaning research on patient records, or other database data. Therefore, ethical review was not necessary nor was required beforehand this study. However, the data gathering and analysis were performed with research permissions granted by institutional review boards of Turku and Tampere University Hospitals.

## Supporting information

Supplementary Material

## Data Availability

Patient health records are confidential information and cannot be made publicly available. However, access to the datasets can be applied from Auria Clinical Informatics and Tays Research Services, with data permits granted by the Hospital District of Southwest Finland and the Pirkanmaa Hospital District, respectively.
