# Supplementary material for: Improved risk prediction of chemotherapy‐induced neutropenia—model development and validation with real‐world data
Source: Cancer Med. 2021 Dec 3;11(3):654–63. doi: 10.1002/cam4.4465 (PMC8817096; doi:10.1002/cam4.4465)
Supplement: Supplementary file 1 — Supplementary Material [file CAM4-11-654-s001.docx]

Improved risk prediction of chemotherapy-induced neutropenia – model development and validation with real-world data

Mikko S. Venäläinen, Eetu Heervä, Outi Hirvonen, Sohrab Saraei, Tomi Suomi, Toni Mikkola, Maarit Bärlund, Sirkku Jyrkkiö, Tarja Laitinen, Laura L. Elo

This document includes Supplementary Tables 1-4 and additional details related to methodology, characteristics of the validation cohort, results obtained using a model developed using conventional stepwise variable selection procedure, and a description how to use the developed Lasso model in practice.

# Methods

## Ethics, data gathering, analysis environment and confidentiality

The data gathering and analysis were performed with the research permission granted by institutional review boards of Turku and Tampere University Hospitals. The real-world data was gathered by Auria and Tays Clinical Informatics teams. Raw patient data is collected from multiple operative systems into the hospital data lake, and then harmonized, validated and curated for secondary use in close collaboration with local clinicians. Each data release for research projects is logged in accordance with the Finnish data privacy legislation and EU General Data Protection Regulation. The pseudonymized research data was analyzed separately in both hospitals using in-house data analysis platform to maximize the privacy and security of the study subjects' data, enforcing two-level authentication and user logging. The data set was archived within the hospital platform for later reference.

## Study endpoint and candidate predictors

The intravenous treatment regimens were grouped based on their mechanism of action as alkylating agents (cyclophosphamide, dacarbazine, ifosfamide, procarbazine, streptozotocin), taxanes (cabazitaxel, docetaxel, paclitaxel), anthracyclines (doxorubicin, epirubicin), antimetabolites (fluorouracil, gemcitabine, methotrexate, pemetrexed), antitumor antibiotics (bleomycin, mitomycin, mitoxantrone), microtubule inhibitors (eribulin), platinum (cis-, carbo- and oxaliplatin), topoisomerase inhibitors (etoposide, irinotecan, topotecan), vinca alkaloids (vinblastine, vincristine, vinflunine), and monoclonal antibodies (trastuzumab, bevacizumab, cetuximab and panitumumab). The G-CSFs included filgrastim (multiple manufacturers, including filgrastim biosimilars), pegfilgrastim, and lipegfilgrastim.

In addition to the independent predictors (Table 1), we considered interaction terms between different intravenous treatment regimens and interactions between metastasis and the different cancer categories as candidate predictors.

## Model development

Penalized regression is an effective method for constructing accurate and generalizable risk prediction models, especially when the number of events as compared to the number of predictors is low [1]. Here, penalized logistic regression with least absolute shrinkage and selection operator (Lasso) penalty was used and the model performance and generalizability were optimized using 5-fold cross-validation. To account for the effect of random subsampling during cross-validation and resulting model variability, the model development was performed in multiple steps. First, the feature space was reduced by repeating the model construction and cross-validation 100 times and selecting the initial set of variables with non-zero influence. Next, the same process was repeated to rank the remaining variables based on the frequency of their occurrence in the models. Finally, variables with occurrence rate of over 50% were selected for training the final model.

## Risk prediction using penalized and standard logistic regression

After fitting a logistic regression model, individualized risk estimates based on the included variables can be obtained as the inverse logit of the difference between the model intercept (constant term) and the raw risk score as:

$$Risk (\%)=\frac{1}{1+exp(-\left( Intercept-Raw score \right))}\times100\%$$

where raw risk score is calculated as a sum of patient-specific risk factors weighted with the regression coefficients. The key difference between a penalized regression model, such as the Lasso, and a standard regression model is that in penalized regression some of the regression coefficients may be set to zero meaning that they are not required when making predictions. This leads to simpler models that are easier to apply in practice.

## Comparison against previously introduced models

We compared the predictions of the Lasso model against the predictions obtained using the Lyman model [2] as well as the revised version of the Lyman model introduced by Li *et al.* [3], referred to as the Lyman model and the Li model, respectively. We used the simplified version of the Li model, without the newly identified comorbidities, since they were reported not to improve the performance in terms of AUROC [3]. For both models, the risk predictions were obtained by converting the reported odds ratios to regression coefficients using natural logarithm transformation [2,3]. The originally reported odds ratios are summarized in Supplementary Table 1. The constant term reported for the Lyman model was -3.423 but for the Li model, the intercept was not reported. Therefore, the intercept was chosen so that all risk predictions were strictly above 0% and below 100%. When evaluating the performance of the risk prediction model in terms of area under the receiver operating characteristic curve, the intercept does not play as central role as the receiver operating characteristic curve considers all possible classification thresholds, regardless of the mean level of risk.

## Comparison against conventional stepwise variable selection procedure

To demonstrate the benefit of penalized regression over conventional variable selection approaches, we developed an additional risk prediction model for neutropenic infection using the stepwise procedure implemented in the function *stepAIC* in R package *MASS* (version 7.3-49). We used option “both” for direction argument which enables the method to start with the full model and then performs both backward deletion and forward inclusion to find an optimal set of variables for a conventional logistic regression model. The training material was the same as for the Lasso model.

**Supplementary Table 1.** The odds ratios reported for previously introduced models for multiple cancer types by Lyman et al. [2] and Li et al. [3]. Prior use, the odds ratios were first converted to the regression coefficients via natural logarithm transformation.

| **Variable** | **Lyman model** | **Li model** | **Additional information** |
| --- | --- | --- | --- |
| **Basic information** |  |  |  |
| Age≥65 years | 1.297 | 1.16 |  |
| Prior chemotherapy | 1.925 | - | All patients in our study were on their first cycle of chemotherapy |
|  |  |  |  |
| **Baseline laboratory values** |  |  |  |
| AST>35 U/l | 1.422 | - |  |
| Alkaline phosphatase>120 U/l | 1.469 | - |  |
| Bilirubin>1 mg/dl | 2.152 | - | Values were first converted from μmol/l to mg/dl using factor 1/17 |
| GFR ml/min | 0.993 | 1.00 | Estimated using CKD-EPI creatinine equation [4] implemented in R package *nephro* |
| WBC ×10^3^/mm^3^ | 0.930 | 1.03 |  |
|  |  |  |  |
| **Cancer type** |  |  |  |
| Breast | 0.842 | 2.00 |  |
| Small cell lung | 1.556 | 2.14 |  |
| Non-small cell lung | 0.594 | 0.62 |  |
| Ovarian | 0.515 | 0.42 |  |
| Colorectal | 1.00 | 1.00 |  |
| Lymphoma | 0.510 | - | Not included in our study |
| NHL | - | 4.38 | Not included in our study |
| Gastric | - | 3.66 |  |
|  |  |  |  |
| **Medication** |  |  |  |
| Immunosuppressives | 1.554 | 1.40 |  |
| Primary CSF prophylaxis | 0.120 | - |  |
| Planned RDI≥85% | 2.018 | 1.37 |  |
|  |  |  |  |
| **Chemotherapy** |  |  |  |
| Alkylating agents | 5.853 | 0.64 |  |
| Anthracyclines | 7.353 | 1.37 |  |
| Gemcitabine | 3.092 | 3.45 |  |
| Platinum(s) | 1.830 | 0.97 |  |
| Taxanes | 2.850 | 4.79 |  |
| Topoisomerase I inhibitors | 18.579 | 2.13 |  |
| Topoisomerase II inhibitors | 8.815 | 2.31 |  |
| Vinorelbine | 4.218 | 2.05 |  |
| Nucleotide analogs and precursor analogs | - | 0.70 |  |
| Vinca alkaloids | - | 1.65 |  |
| Targeted | - | 0.99 |  |
| Others (DNA cross‐linkers, epothilones, and immunomodulators) | - | 5.97 | Not included in our study |
| AST = aspartate aminotransferase, GFR = glomerular filtration rate, WBC = white blood cell count, NHL = non‐Hodgkin's lymphoma, CSF = colony-stimulating factor, RDI = relative dose intensity | | | |

# Results

## Tampere University Hospital validation cohort

Supplementary Table 2 summarizes the characteristics of the validation cohort from Tampere University Hospital.

**Supplementary Table 2.** Characteristics of the Tampere University Hospital cohort according to the occurrence of neutropenic infection during the first round of chemotherapy.

|  |  | **Neutropenic infection** | | **Neutropenic infection** | | ***P* value^‡^** |
| --- | --- | --- | --- | --- | --- | --- |
|  |  | **No** | | **Yes** | |  |
|  |  | **N=4421 (%)** | | **N=173 (%)** | |  |
| **Demographics** | |  |  |  |  |  |
| Sex, n (%) | |  |  |  |  | <0.001 |
|  | Male | 1735 | (39) | 36 | (21) |  |
|  | Female | 2686 | (61) | 137 | (79) |  |
| Age | |  |  |  |  | <0.001 |
|  | <40 | 251 | (6) | 16 | (9) |  |
|  | 40-65 | 2020 | (46) | 100 | (58) |  |
|  | >65 | 2150 | (48) | 57 | (33) |  |
| Mean BMI, kg/m² (standard deviation) | | 26.6 | (7.4) | 26.9 | (5.0) | 0.3 |
| Mean body surface area, m² (standard deviation) | | 1.9 | (0.2) | 1.8 | (0.2) | 0.2 |
|  | |  |  |  |  |  |
| **Use of prophylactic G-CSFs,** n (%) | | 205 | (5) | 16 | (9) | 0.009 |
|  | |  |  |  |  |  |
| **Comorbidities [ICD-10], n (%)** | |  |  |  |  |  |
| COPD [J44, J96] | | 168 | (4) | 7 | (4) | 1.0 |
| Coronary heart disease [I25] | | 194 | (4) | 5 | (3) | 0.4 |
| Diabetes [E10-E14] | | 538 | (12) | 10 | (6) | 0.02 |
| Heart failure [I50] | | 71 | (2) | 5 | (3) | 0.3 |
| Renal impairment [N17-N19] | | 58 | (1) | 4 | (2) | 0.4 |
| Liver failure [K70-K75] | | 14 | (1) | 1 | (1) | 1.0 |
| Rheumatoid arthritis [M05-M07] | | 72 | (2) | 1 | (1) | 0.4 |
| Ulcer disease [K25-K27] | | 44 | (1) | 0 | (0) | 0.4 |
|  | |  |  |  |  |  |
| **Metastatic disease** | |  |  |  |  |  |
| C77-C79 detected, n (%) | | 1393 | (32) | 37 | (21) | 0.006 |
|  | |  |  |  |  |  |
| **Laboratory test results, mean (standard deviation)** | |  |  |  |  |  |
| Absolute neutrophil count [×10^9^/l] | | 4.7 | (2.8) | 4.5 | (2.6) | 0.2 |
| Alanine aminotransferase [U/l] | | 29.4 | (35.7) | 26.2 | (22.7) | 0.3 |
| Alkaline phosphatase [U/l] | | 98.0 | (112.5) | 88.6 | (107.9) | 0.001 |
| Average red blood cell size [fl] | | 89.2 | (5.5) | 90.4 | (4.8) | 0.001 |
| Blood hematocrit [%] | | 39.4 | (4.2) | 39.8 | (4.1) | 0.2 |
| Blood hemoglobin [g/l] | | 130.5 | (15.6) | 132.8 | (13.7) | 0.06 |
| Hemoglobin amount per red blood cell [pg] | | 29.6 | (2.2) | 30.3 | (1.8) | <0.001 |
| Leukocyte count [×10^9^/l] | | 7.5 | (3.1) | 7.1 | (2.8) | 0.06 |
| Plasma bilirubin [μmol/l] | | 8.8 | (7.3) | 9.5 | (8.5) | 0.4 |
| Plasma potassium [mmol/l] | | 4.0 | (0.4) | 4.0 | (0.3) | 0.06 |
| Plasma sodium [mmol/l] | | 139.2 | (3.5) | 139.4 | (3.7) | 0.3 |
| Red blood cell count [×10^12^/l] | | 4.4 | (0.5) | 4.5 | (0.4) | 0.3 |
| Serum creatinine [μmol/l] | | 72.1 | (26.6) | 68.7 | (15.1) | 0.07 |
| Thrombocyte count [×10^9^/l] | | 300.4 | (107.3) | 294.9 | (95.7) | 0.6 |
|  | |  |  |  |  |  |
| **Planned relative dose intensity** | |  |  |  |  | <0.001 |
|  | <85% | 1089 | (27) | 12 | (7) |  |
|  | ≥85% | 2967 | (73) | 162 | (93) |  |
|  | |  |  |  |  |  |
| **Intravenous treatment regimens, n (%)** | |  |  |  |  |  |
| Alkylating agents | | 489 | (11) | 28 | (16) | 0.05 |
| Anthracyclines | | 207 | (5) | 14 | (8) | 0.06 |
| Antimetabolites | | 1626 | (37) | 21 | (12) | <0.001 |
| Antitumor antibiotics | | 105 | (2) | 5 | (3) | 0.9 |
| Monoclonal antibodies | | 561 | (13) | 31 | (18) | 0.06 |
| Platinum | | 1881 | (43) | 49 | (28) | <0.001 |
| Taxanes | | 1818 | (41) | 122 | (71) | <0.001 |
| Topoisomerase inhibitors | | 250 | (6) | 20 | (12) | 0.002 |
| Vinca alkaloids | | 159 | (4) | 3 | (2) | 0.3 |
|  | |  |  |  |  |  |
| **Cancer group [ICD-10],** n (%) | |  |  |  |  | <0.001 |
|  | Breast [C50] | 1360 | (31) | 101 | (58) |  |
|  | Central nervous system [C70-72] | 175 | (4) | 1 | (1) |  |
|  | Colorectal [C18-20] | 655 | (15) | 2 | (1) |  |
|  | Female reproductive [C51-57] | 414 | (9) | 16 | (9) |  |
|  | Gastric [C15-16] | 220 | (5) | 5 | (3) |  |
|  | Head and neck [C00-14, C30-32] | 321 | (7) | 2 | (1) |  |
|  | Lung, non-small cell [C33-35] | 311 | (7) | 8 | (5) |  |
|  | Lung, small cell [C33-35] | 91 | (2) | 8 | (5) |  |
|  | Melanoma [C43] | 21 | (1) | 0 | (0) |  |
|  | Other gastrointestinal [C17, C21, C22, C26] | 66 | (2) | 1 | (1) |  |
|  | Pancreas and gallbladder [C23-25] | 277 | (6) | 4 | (2) |  |
|  | Prostate [C61] | 181 | (4) | 7 | (3) |  |
|  | Sarcoma [C40-41, C46-49] | 96 | (2) | 12 | (7) |  |
|  | Testicular [C62] | 104 | (2) | 4 | (2) |  |
|  | Urinary tract [C65-68] | 61 | (1) | 1 | (1) |  |
|  | Other^†^ | 68 | (2) | 1 | (1) |  |
| † Category includes all remaining ICD-10 codes from C00-79. | | | | | | |
| ‡ Comparisons between the groups either having or not having NI were tested using the Mann-Whitney test for continuous variables and the chi-squared test or Fisher’s exact test (N<5) for categorical variables. | | | | | | |

## Performance of the model developed using the stepwise method

The variables identified for the risk prediction model developed using the stepwise variable selection procedure have been summarized in Supplementary Table 3. Although the discrimination performance of the stepwise model was comparable to the Lasso model in the Turku University Hospital training cohort (AUROC 0.83, 95% CI: 0.80–0.86 vs AUROC 0.84, 95% CI 0.81–0.86, P=0.54) where it also outperformed both Lyman (P<0.001) and Li (P<0.001) models, the performance was significantly poorer in the Tampere University Hospital external validation cohort as compared to the Lasso model (AUROC 0.70, 95% CI: 0.65–0.76 vs AUROC 0.75, 95% CI: 0.69–0.77, P=0.02). However, it outperformed the Lyman model (P<0.001) and achieved performance comparable to the Li model (P=0.99). Furthermore, the stepwise model required the use of eight additional variables compared to the Lasso model, limiting its use in clinical practice.

**Supplementary Table 3.** Risk prediction model for neutropenic infection obtained using the stepwise variable selection procedure. Positive coefficient indicates risk-increasing effect whereas negative coefficient indicates risk-reducing effect.

| **Covariate** | **Coefficient** |  | **Covariate** | **Coefficient** |
| --- | --- | --- | --- | --- |
| *Demographics* |  |  | *Laboratory test results* |  |
| Mean body surface area | 1.175 |  | Neutrophil count [×10^9^/l] (per ln increase) | -0.550 |
|  |  |  | Thrombocyte count [×10^9^/l] (per ln increase) | -1.290 |
| *Comorbidities* |  |  |  |  |
| COPD | 1.704 |  | *Treatment regimen* |  |
| Diabetes | -1.466 |  | Use of taxanes | 1.295 |
|  |  |  | Use of monoclonal antibodies | -16.122 |
| *Cancer type* |  |  | Combined use of taxanes and monoclonal antibodies | 17.146 |
| Breast cancer | 17.533 |  | Use of topoisomerase inhibitors | 3.513 |
| Sarcoma | 20.173 |  | Use of antimetabolites | -1.081 |
| Colorectal | 15.923 |  |  |  |
| Pancreatic | 17.078 |  | *Actions to reduce risk* |  |
| Prostate | 14.764 |  | Use of G-CSF | -1.644 |
| Lung, non-small cell | 16.188 |  | Relative dose intensity <85% | -1.004 |
| Model intercept = -14.787 | | | | |

## Assessing the effect of G-CSFs on predicted NI risk

By considering the use of G-CSF as a modifiable risk factor, the Lasso model enables easy estimation of risk of NI with or without the use of G-CSFs. Supplementary Table 4 shows an example calculation for a breast cancer patient who was treated with taxanes and monoclonal antibodies and had neutrophil count of 3.5×10^9^/l and thrombocyte count of 250×10^9^/l before the first round of treatment. Her risk for NI was more than four times higher without G-CSF (32.8%) than with G-CSF (7.6%).

**Supplementary Table 4.** Risk of neutropenic infection for a breast cancer patient (profiled for given chemotherapy and pre-treatment blood values) treated with or without G-CSF using the developed Lasso model.

| **Covariate** | **Coefficient** | **Example patient value** | **Coefficient × Example patient value (without G-CSF)** | **Coefficient × Example patient value (with G-CSF)** |
| --- | --- | --- | --- | --- |
| *Cancer type* |  |  |  |  |
| Breast cancer | 2.361 | 1 | 2.361 | 2.361 |
| Sarcoma | 3.694 | 0 | 0 | 0 |
|  |  |  |  |  |
| *Laboratory test results* |  |  |  |  |
| Neutrophil count [×10^9^/l] (per ln increase) | -0.282 | 1.253 [=ln(3.5)] | -0.353 | -0.353 |
| Thrombocyte count [×10^9^/l] (per ln increase) | -0.966 | 5.521 [=ln(250)] | -5.333 | -5.333 |
|  |  |  |  |  |
| *Treatment regimen* |  |  |  |  |
| Use of taxanes | 1.262 | 1 | 1.262 | 1.262 |
| Combined use of taxanes and monoclonal antibodies | 0.871 | 1 | 0.871 | 0.871 |
| Use of topoisomerase inhibitors | 3.305 | 0 | 0 | 0 |
| Use of antimetabolites | -0.787 | 0 | 0 | 0 |
|  |  |  |  |  |
| *Actions to reduce risk* |  |  |  |  |
| Use of G-CSF | -1.780 | - | 0 | -1.780 |
| Relative dose intensity <85% | -0.814 | 0 | 0 | 0 |
| Raw score (sum) |  |  | -1.192 | -2.981 |
| **Predicted risk**  $=\frac{1}{1+e^{-(0.477+Raw score)}}$ |  |  | **Without G-CSF: 32.8%** | **With G-CSF: 7.6%** |

# References

[1] Pavlou M, Ambler G, Seaman SR, Guttmann O, Elliott P, King M, et al. How to develop a more accurate risk prediction model when there are few events. BMJ 2015;351:h3868. https://doi.org/10.1136/bmj.h3868.

[2] Lyman GH, Kuderer NM, Crawford J, Wolff DA, Culakova E, Poniewierski MS, et al. Predicting individual risk of neutropenic complications in patients receiving cancer chemotherapy. Cancer 2011;117:1917–27. https://doi.org/10.1002/cncr.25691.

[3] Li Y, Family L, Chen LH, Page JH, Klippel Z, Xu L, et al. Value of incorporating newly identified risk factors into risk prediction for chemotherapy-induced febrile neutropenia. Cancer Medicine 2018;7:4121–31. https://doi.org/10.1002/cam4.1580.

[4] Inker LA, Schmid CH, Tighiouart H, Eckfeldt JH, Feldman HI, Greene T, et al. Estimating Glomerular Filtration Rate from Serum Creatinine and Cystatin C. New England Journal of Medicine 2012;367:20–9. https://doi.org/10.1056/NEJMoa1114248.
